# Self-care strategies used by disaster responders after the 2023 earthquake in Turkey and Syria: a mixed methods study

**DOI:** 10.1186/s12873-024-01105-8

**Published:** 2024-10-17

**Authors:** Karin Blomberg, Jason Murphy, Karin Hugelius

**Affiliations:** 1 Faculty of Medicine and Health, Örebro university, Örebro, SE-701 82 Sweden; 2 Department of Surgical and Perioperative Sciences, Umeå University, Umeå, Sweden; 3 The Red Cross University College, Stockholm, Sweden

**Keywords:** Self-care, Health promotion, Disaster responders, Disaster recovery, Survey, Resilience

## Abstract

**Background:**

Disaster responders are exposed to several physical and mental health risks. This study aimed to describe self-care strategies used by disaster responders after the earthquake in Syria and eastern Turkey in February 2023.

**Methods:**

A study specific web-based questionnaire survey was used to collect quantitative and qualitative data according to a convergent mixed methods approach. Data from 252 disaster responders responding to the earthquakes in Turkey and Syria were analyzed using both descriptive and analytical statistics and summative content analysis of free-text answers. Data were collected in March to July, 2023.

**Results:**

The most used self-care strategies included resting, social support from colleagues in the field, extra intake of food or drink, and intake of medicines. The recovery strategies varied due to previous disaster response experience, indicating that supportive self-care strategies can be developed or learned.

**Conclusion:**

Given the extreme conditions and limited possibilities of external support, sufficient self-care is an essential competence among disaster responders. Self-care strategies can be both external processed such as intake of medicines, social support from others, and internal processes such as personal reflection. Providing oneself with self-care activities seems to be a skill developed with increasing experience supported by pre-deployment training. Therefore, to enhance resilience, self-care strategies should be encompassed in pre-disaster response training.

**Supplementary Information:**

The online version contains supplementary material available at 10.1186/s12873-024-01105-8.

## Background

The well-being of disaster responders is an essential component of disaster relief. However, both physical and mental health problems have been reported among disaster responders, both during the response and after [1, 2]. In addition, disaster responders and humanitarian aid workers are being confronted with increasing security risks that might lead to weapons attacks, hostage situations, or other security threats [3]. The central purpose of crisis management is to mitigate the negative effects of disaster events [4], and if disaster responders cannot provide their support and expertise to the affected population, the effects of a disaster might be even worse.

Disaster responders are at a high risk of experiencing both physical and mental health problems after a disaster deployment [5] that can also have long-term negative effects after the deployment [5, 6]. Physical health problems range from non-serious to life-threatening infections, trauma, or cardiovascular events [1, 7, 8]. Mental health problems range from normal stress reactions to psychopathological conditions. The prevalence of post-traumatic stress disorder among disaster responders has been reported to range from 0 to 34% and depression from 21 to 53% [9]. Nevertheless, these numbers are generally lower for disaster responders compared with the affected population in general [10]. Despite these risks of suffering from mental health problems due to extreme events like a disaster, it is important to emphasize that most people affected by severe stress, both the primarily affected and disaster responders, recover without any specific interventions [11, 12]. Stress resilience means that affected individuals maintain balance and healthy functioning, sometimes referred to as a stable trajectory of healthy functioning [13], during and after highly adverse events [14]. Resilience can be seen as a recovery process itself or as the goal of such a process, or both [13]. The exact strategy or process to enable resilience after stressful events is still not fully known, and the determinants of resilience differ depending on the context and the individual affected. However, given the context of a disaster, where the demands are high, the level of uncertainty is high, and there is limited availability of medical or psychosocial professional care, self-care strategies become even more essential to cope with extreme stress and perceived health problems. Therefore, the well-being and self-care strategies to manage the demands that come with disaster response missions are essential not only for the disaster responders as individuals but also for the affected population.

Self-care can be defined as “the ability to care for oneself through awareness, self-control, and self-reliance in order to achieve, maintain, or promote optimal health and well-being.” [15]. Similar terms used include self-management, self-monitoring, and self-help [16]. The concept of self-care has a long history, relying mainly on Dorotea Orme´s self-care theory [17], which describes self-care activities as a natural instinct to do things that enhance perceived health, quality of life, and dignity despite illness or injury. However, an important component of effective self-care is understanding what threatens well-being and having both knowledge and the capability to provide self-care [17]. Effective self-care strategies have been described as essential components to enhance resilience [14] and promote growth, mastery, and self-efficacy after experiencing negative events such as a disaster [18]. In a disaster context, the possibilities of receiving professional support, medical or psychosocial, are often limited. Therefore, effective self-care strategies are essential for disaster responders to enhance their well-being and provide relief to the people affected.

Despite the increasing awareness of health risks among disaster responders, there are still few studies on what self-care strategies are used by such responders and whether these differ due to experience and other circumstances. Such knowledge is necessary to design appropriate preparations before being deployed to a disaster area, as well as to understand how self-care strategies can be supported or introduced by employers [19]. Therefore, this study aimed to investigate the self-care strategies used by disaster responders after the earthquake in Syria and eastern Turkey in February 2023 and the potential differences between inexperienced and experienced disaster responders and between male and female responders.

## Methods

### Design

A convergent, parallel mixed methods study [20] was conducted, combining statistical analysis of quantitative data and analysis of free-text answers from a web-based survey.

### Study context

In February 2023, an earthquake with a magnitude of 7.8 struck the southeast parts of Turkey and Syria (GLIDE number EQ-2023-000015-TUR and EQ-2023-000015-SYR). The disaster caused a severe humanitarian situation, with about 60,000 deceased and over 115,000 injured persons [21]. Following the earthquake, a massive response was initiated, including both national and international responders, such as urban search and rescue (USAR) teams, emergency medical teams (EMT), civil protection teams, and humanitarian aid workers.

### Study participants, questionnaire, and data collection

A study-specific, web-based questionnaire developed by the authors was used to collect data. The questionnaire comprised multiple-choice questions on health problems and strategies used to manage these, including self-care strategies (see Supplementary file). Additionally, two free-text questions were included for deeper descriptions of the self-care strategies used and for other experiences or comments on self-care or recovery processes that study participants wanted to share. The questionnaire was available in English or Turkish for the participants’ choice. The questionnaire was piloted among 10 Swedish aid workers with experience in disaster response missions. Minor changes in the design and wording were made after the pilot test. No psychometric evaluation was performed. In this paper, data related to self-care strategies are reported. Data on perceived health problems, gained from the same survey, have been reported elsewhere [22].

Study invitations were disseminated to potential study participants by sending the invitation to international organizations (including the International Federation of the Red Cross, Médecins Sans Frontières, Save the Children, United Nations Office for the Coordination of Humanitarian Affairs, World Food Program, United Nations Children’s Fund, the World Health Organization’s EMT Secretariate, and the International Search and Rescue Advisory Group Secretariate) and several national non-governmental organizations and professional response teams. The invitation was sent out eight weeks after the earthquake and was open from March 24 to July 30, 2023. To participate in the study, the participants should have been actively involved in disaster response, be at least 18 years old, and be able to respond to the questionnaire in either English or Turkish.

### Analysis

Descriptive and analytic statistics were conducted. Comparisons between groups, such as gender and experienced compared to non-experienced responders, were analyzed using chi-square tests. A *p*-value of ≥ 0.05 was considered significant. SPSS (IBM Corp. Released 2021. IBM SPSS Statistics for Windows, Version 28.0. Armonk, NY: IBM Corp) was used to analyze the quantitative data. Internal dropouts were not included in the analysis, and specific dropout analyses were not conducted.

The free-text answers were analyzed in accordance with summative content analysis [23] by sorting the words and expressions after their manifest content. Thereafter, the words and expressions were sorted into themes in relation to self-care strategies used and how such were achieved. The number of times specific words or expressions were used was also used to identify patterns and to create contextual meaning of the analysis [23]. One researcher (KH) conducted the primary analysis of the qualitative data analysis, which was thereafter discussed and validated by the research team. In accordance with the convergent, parallel mixed methods approach [20], the quantitative and qualitative results were merged during the analysis phase and the results were presented together. Quotations were used to illustrate the findings.

## Results

Overall, 525 disaster responders participated in the study. Of these, 360 (69%) were male, and 165 (31%) were female, with a mean age of 42 years (ranging from 22 years to 65 years, SD = 10.5). Of the participants, 93 (17%) were national responders, and 432 (80%) were international responders. For more demographics, see Table 1. Thirty-three study participants had written short free-text answers, covering in all about one page of text. The study participants presenting free-text answers were 19 females (58% of all participants presenting free-text answers) and 15 males (42%), with a median age of 40 (mean = 43). Their field of expertise in the mission varied from humanitarian aid (*n* = 9, 27%), management or coordination (*n* = 8, 24%), medical/health (*n* = 6, 18%), and other (*n* = 10, 30%). The majority (*n* = 23, 70%) were international responders. Their experiences ranged from no previous experience (*n* = 19, 58%) to 15 missions (*n* = 1, 3%).

**Table 1:** Study participants’ demographics

		Total *N* = 525 *n* (%)	Male *n* = 358 *n* (%)	Female *n* = 165 *n* (%)	p*	Responders without disaster experience *n* = 279 *n* (%)	Responders with disaster XXXexperience *n* = 263 *n* (%)	*p* *
**Marital status**	Married	312 (59%)	237 (66%)	75 (45%)	< 0.001	144 (52%)	168 (64%)	< 0.001
**Parenthood**	Yes, have children	252 (48%)	219 (61%)	33 (20%)	< 0.001	126 (45%)	126 (48%)	0.075
**National or international**	National responder	93 (18%)	51 (11%)	42 (25%)	0.002	57 (20%)	36 (14%)	0.052
	International responder	432 (80%)	309 86%	123 (75%)	0.002	222 (80%)	210 (48%)	0.087
**Field of expertise in the mission**	Medical or health**	156 (30%)	81 (52%)	75 (48%)	0.087	105 (38%)	51 (19%)	< 0.001
	Urban Search and Rescue	123 (23%)	114 (93%)	9 (7%)	< 0.001	102 (37%)	21 (8%)	< 0.001
	Management/coordination	87 (16%)	72 (83%)	15 (17%)	< 0.001	18 (6%)	69 (28%)	< 0.001
	Other	48 (9%)	15 (31%)	33 (69%)	0.092	12 (4%)	36 (14%)	< 0.001
	Mental health and psychosocial support	36 (7%)	9 (25%)	27 (75%)	< 0.001	21 (8%)	15 (6%)	0.876
	Needs assessment	24 (4%)	6 (25%)	18 (75%)	< 0.001	0%	24 (10%)	N/A
	Nutrition	21 (4%)	18 (86%)	3 (14%)	< 0.001	9 (3%)	12 (5%)	< 0.001
	Shelter	18 (3%)	18 (100%)	0 (0%)	N/A	9 (3%)	9 (2%)	0.654
	Water and sanitation	9 (2%)	6 (67%)	3 (33%)	< 0.001	0 (0%)	9 (4%)	N/A

*Chi-square test. Missing data and “I don´t know or does not want to answer” are not included in the analysis

** Including emergency medical teams

In the entire study sample, the number of previous disaster response mission experiences varied from zero to 15 (mean = 2.4, median = 1.0, SD = 2.6), and about half of the sample (*n* = 279, 52%) had no previous disaster response experience. Of these, 57 (20%) were local responders, and 222 (80%) were international responders. Among those responders who had experiences from six missions or more, 39 (93%) were international responders. Overall, 244 (47%) of all study participants reported some kind of health problems during or after their mission. Furthermore, less than half of the study participants, 213 (41%), had received pre-deployment training on health risks during a disaster response mission (see Table 2).

**Table 2:** Perceived health problems, pre-deployment training on health risks, and strategies used to manage health problems

		Total *N* = 525 *n* (%)	Male *n* = 358 *n* (%)	Female *n* = 165 *n* (%)	p*	Responders without disaster experience *n* = 279 *n* (%)	Responders with disaster experiences *n* = 263 *n* (%)	p*
**Pre-deployment training on health risks**		213 (41%)	141 (39%)	72 (44%)	0.261	60 (22%)	153 (58%)	< 0.001
**Perceived health problems**	Any health problem	244 (45%)	155 (43%)	89 (54%)	0.024	121 (43%)	123 (47%)	0.160
	Mental health problems	196 (36%)	119 (33%)	77 (47%)	0.003	88 (32%)	108 (41%)	0.025
	Physical health problems	201 (37%)	147 (41%)	54 (33%)	0.082	105 (38%)	96 (37%)	0.790
**Self-care used**	Self-care used	279 (53%)	174 (48%)	105 (64%)	<,001	156 (56%)	123 (47%)	0.404

*Chi-square test. Missing data and “I don´t know or does not want to answer” are not included in the analysis

The qualitative analysis rendered two themes; (I) self-care strategies used, including the codes physical activities, social support and time and reflection, and (II) pre-deployment health risk training, previous experience, and self-care strategies, where no codes were identified.

### Self-care strategies used

Most of the study participants (*n* = 279, 53%) had used self-care strategies during or after their assignments. Women employed self-care significantly more than men (*p* = .001), but no significant difference between experienced and inexperienced responders could be observed (see Table 2). In the free-text answers, the study participants expressed several ways of recovering from their deployment. Some stated that they had not yet recovered or doubted that they would.

*I have not recovered. I will never forget. I dream still.*

The most-used self-care strategy to recover from health problems during or after the deployment included resting, social support from colleagues, extra intake of food or drink, and intake of prescribed or unprescribed medicines. Social support from family or friends was used to a lesser extent (see Table 3).

**Table 3:** Self-care strategies used by responders

		Total *N* = 252	Male *n* = 358	Female *n* = 165	p*	Responders without disaster experience *n* = 279	Responders with disaster experiences *n* = 263	P*
**Used any kind of self- care**	**Yes**	279 (52%)	174 (49%)	105 (64%)	< 0.001	156 (56%)	123 (47)	0.760
**Type of self-care used**	**Rested**	123 (23%)	75 (21%)	48 (29%)	0.776	60 (22%)	63 (24%)	0.390
	**Social support from colleagues**	102 (19%)	54 (15%)	48 (29%)	0.029	57 (20%)	59 (22%)	0.045
	**Medicines**	57 (11%)	39 (11%)	18 (11%)	0.086	21 (8%)	36 (14%)	0.029
	**Extra intake of food and/or drinks**	57 (11%)	39 (11%)	18 (11%)	0.086	30 (11%)	27 (10%)	0.378
	**Social support from family or friends**	33 (6%)	21 (6%)	12 (7%)	0.702	12 (4%)	21 (8%)	0.035
	**Change of duties**	24 (4%)	9 (3%)	15 (9%)	0.025	12 (4%)	12 (5%)	0.586
	**Other**	21 (4%)	9 (3%)	12(7%)	0.104	12 (4%)	9 (3%)	0.323

*Chi-square test. Missing data and “I don´t know or does not want to answer” are not included in the analysis

Physical activities.

Several participants noted that physical exercise, such as sports, running, or taking a walk in nature, was a recovery strategy for them. Especially being in the nature was mentioned as supportive. Sometimes, physical exercise was combined with, for example, listening to music.

*I walk and listen to music.*

Yoga and meditation were also reported as used self-care strategies, individually or combined with other physical activities.

Social support.

Social support was a commonly used self-care strategy. Social support from colleagues was used to a significantly larger extent among experienced responders compared to responders with no previous experience and used more by females than males (see Table 3). Social support from disaster response colleagues, such as team members, was considered as the most important support.

*I think the most important people are your teammates*,* you should really rely on them. You don’t have to say much*,* just stay together.*

Some study participants highlighted the importance of leaving the disaster area together and keeping in contact with field team colleagues to facilitate the recovery process.

Time and reflection.

Returning home to a safe place was part of the recovery strategy. Before returning to a safe place, the recovery process could not start. After coming to a safe place, allowing time to heal was essential in this process.

*I take my time to think about things*,* to be with friends*,* to relax. Things usually gets better.*

Time allowed the responder to decrease physical and mental stress reactions and to reflect on their experiences. The recovery process took more time than expected, and some study participants mentioned that the did not have enough time to reflect and recover. Some participants also used prayer as a self-care strategy.

### Pre-deployment health risk training, previous experience, and self-care strategies

The strategies used varied depending on previous experiences from being deployed to disaster areas, where more experienced responders used medications and social support from colleagues to a larger extent compared to responders with no previous experience (see Table 3). The study participants expressed that self-care was something they had learned by experience.

*You must plan for rest and recovery period. It takes some days*,* and you cannot come around it*,* you just need to adapt. You will learn how to do it and what you need by experience.*

Overall, 114 (22%) of all study participants had received pre-deployment training on health risks. Of these, responders who had received this training used self-care to a larger extent (*n* = 102, 90%) compared to responders who had not received such training or were unsure if they had received such training (16%, *n* = 18, *p* = < 001). Furthermore, responders with academic education used more self-care strategies than responders with no academic education (*p* = .010) Moreover, medical personnel used more self-care strategies than non-medical responders (*p* = .010).

## Discussion

A broader variety of self-care health strategies were used to reduce health problems during and after a mission and were, in some aspects, correlated to gender, disaster experience, and pre-deployment training on health risks, and the recovery process required substantial time after the mission.

The most reported self-care strategy in this survey was “resting.” However, it was not defined in the survey what resting meant. The free-text answers imply that resting included a change in environment, such as spending time in nature, but also that resting required sufficient time to process the impressions and move from one state to another. These findings raise a question on what “resting” is in relation to being deployed in a disaster means and imply that “resting” is subjective and unique to the individual. The dictionary definition of resting is “to stop doing a particular activity or stop being active for a period of time in order to relax and get back your strength.” [24]. That is surely possible for international responders participating in short-term missions in another country. However, national responders do not share this opportunity since the disaster may imply personal losses. Furthermore, national responders’ ability to “rest” may be constrained due to an inability to physically detach from human suffering and the environmental challenges caused by the incident. To fully understand the meaning, various forms, requirements, and effects of “resting” in terms of resilience or recovery processes among disaster responders, further studies are needed.

Resilience is a commonly used term to describe how an individual or organization “bounce[s] back” after adversity [25, 26]. However, it should be noted that resilience does not necessarily imply the ability to not experience stress, such as intrusive memories, being emotional, or feeling distressed, but rather the ability to carry on with life [14, 27]. The concept of psychological resilience is constantly debated; however, a common consensus is that resilience should be seen as a dynamic process of the adaption of stress, involving both personal traits and organizational structures [27]. In response to stress, individuals who confront their fears, maintain an optimistic worldview, tend to use social support, and rely on their inner moral compass seem to be more resilient [14]. In addition, individuals who accept personal responsibility for their own well-being are more likely to follow a resilient trajectory [14]. This resilience perspective emphasizes the importance of disaster responders having access to effective self-care strategies to stay resilient and cope with the demands that a disaster implies.

To mitigate psychological stress and mental health problems, the power of social support is well acknowledged [28, 29]. Therefore, the finding that social support from colleagues was one of the most frequently used recovery strategies, increasing with the number of missions, is not surprising. Social support should be informal and naturally occurring to benefit disaster responders’ resilience [30], and responders who experience social support may require a shorter recovery time [31]. However, a large variance in psychological outcomes has also been reported among disaster responders with perceived good social support [30]. When trying to develop and implement interventions aimed at supporting disaster responders’ mental health, organizational, social, personal, and psychological factors may be crucial [32]. Given the well-known lack of evidence for early interventions, such as psychological debriefing or group counseling, such interventions cannot be recommended on a routine basis [33–35]. Since responders with the highest burden of mental health problems are least likely to request or receive professional support [36], both the promotion of well-functioning teams offering informal social support to all members and formal structures to monitor the responders and offer professional support, sometimes referred to as a “screen and treat” strategy, are recommended [37].

One important question is whether disaster responders can develop or train their self-care or resilient strategies over time or whether such processes are primarily personal attributes. Previous studies suggest that it is possible to develop self-care skills with growing professional and personal experiences from similar situations [19]. This study has shown that both previous experiences from other disaster missions and pre-deployment training on health and well-being during such missions may support the development of self-care strategies and resilience among disaster responders. Pre-disaster training that focuses on individual and collegial coping strategies may mitigate the inherent stress of disaster [38, 39]. Referring to the concept of duty of care, providing necessary professional support to prepare for the demands associated with disaster deployment and robust follow-up systems when self-care is insufficient should be obligatory for disaster response organizations.

Despite the obvious importance of effective self-care strategies for disaster responders, scientific interest has so far been low. Web-based psychosocial interventions developed to target disaster-related distress have been developed, but few programs have been formally evaluated [40]. To enhance resilience from a systematic view and for the individual disaster responder, further studies on successful self-care strategies, theories, or models on how such strategies are developed from an individual and an organizations perspective, and the relation in-between these two, on are strongly needed.

### Limitations

This mixed methods study has several limitations. The use of a non-randomized study sample and one disaster event entails a risk of selection bias and reduces the possibility of generalizing the results [41]. However, the disaster context reduces the possibility of conducting randomized sampling procedures [42, 43], and given the study sample includes a great variation of disaster responders, both national and international responders, and a fairly large number of responders compared with other studies on health effects in disasters [44], the results adds value to the field. Since it is not known how long ago the study participants responded to a disaster area in relation to when they answered the questionnaire, there might also be a risk of memory bias. The free-text answers used in the qualitative part of the study were mostly very short and sometimes only consisted of one single word. Therefore, summative content analysis [23] was chosen for the qualitative analysis. This method is recommended for analyse free text answers in surveys [45]. To obtain a deeper understanding of both the use and meaning of self-care among disaster responders and how the skill to develop such strategies can be promoted, in-depth interviews are suggested.

## Conclusions

Given the extreme conditions and limited possibilities for external support that characterize disaster response, sufficient self-care is an essential competence among disaster responders. Self-care strategies may include internal processes, such as reflection, as well as the intake of medicines, social support from others, and sometimes, the proficiency to know when self-care is insufficient. Providing oneself with self-care activities seems to be a skill developed via increasing experience supported by pre-deployment training. Therefore, to enhance resilience, self-care strategies and a reflection on recovery processes should be encompassed in pre-disaster training for disaster responders.

## Electronic supplementary material

Below is the link to the electronic supplementary material.

Supplementary Material 1

## Data Availability

The datasets analysed during the current study are available from the corresponding author on reasonable request.
