# Supplementary material for: Self-care strategies used by disaster responders after the 2023 earthquake in Turkey and Syria: a mixed methods study
Source: BMC Emerg Med. 2024 Oct 17;24:195. doi: 10.1186/s12873-024-01105-8 (PMC11488114; doi:10.1186/s12873-024-01105-8)
Supplement: Supplementary file 1 — Supplementary Material 1 [file 12873_2024_1105_MOESM1_ESM.docx]

Supplementary file: Questionnaire

| **Question** | **Answer alternatives** |
| --- | --- |
| Are you… | Female  Male  Other  I don't want to answer this question |
| How old are you? (in years) | (Number) |
| What is your marital status? | Single (or divorced/separated)  Married or in a domestic partnership  Widowed  Other |
| Do you have children? | No  Yes |
| What is your profession? (in your everyday work) | Medical doctor/physician  Nurse  Medic/paramedic  Rescuer/firefighter  Building engineer  Police officer/military professional  Social work/psychologist  Logistic officer  Information and communication technology officer  Water and sanitation expert  Humanitarian aid worker  Other (please comment below) |
| Did you participate in the response | As part of your ordinary work (e.g., as a nurse or firefighter working every day in the disaster area)  As a local responder in a temporary deployment (e.g., as part of a local medical team deployed to the disaster area)  As an international staff (e.g., as part of an international team) |
| Did you participate in the response as… | Part of your ordinary work or mandatory deployment  Part of your ordinary work or voluntary deployment  Part of your temporary or mandatory deployment  Part of your temporary or voluntary deployment |
| How many disaster response missions have you been deployed in (including this)? | (Number) |
| What was your function/position during this mission? | Emergency medical team (EMT)  Health- or medical (not EMT)  Mental health and psychosocial support  Urban search and rescue (USAR)  Needs assessment  Management, coordination, and logistics (e.g., UNDAC or EUCPT)  Shelter  Food or nutrition  Water and sanitation  Early recovery  Other humanitarian aid  Other |
| How long was your deployment/mission? | 1–7 days  8–21 days  More than 21 days |
| Have you done any preparatory training on health risks or how to stay healthy in disaster response missions? | Yes  No  I don't know |
| Did you experience any of the below health issues during or after your mission? | Yes  No |
| If Yes; Did this health issue require… | Self- care  Professional medical care or psychosocial counselling during the mission/ disaster response in the  field  Medical evacuation from the field (or unplanned end of mission)  Professional medical care or psychosocial counselling after the mission (other than routine health  check up) |
| What kind of self- care strategies did you use?  Other (please comment below) | Medicine/s (prescripted or not)  Extra intake of food and/ or drinks  Change of duties  Rested  Social support from colleagues in the field  Social support from family or friends |
| Please briefly describe your self care strategies with your own words | Free text answer |
| Do you have any other thoughts you would like to share with the research team? | Free text answer |
